# Subcutaneous Emphysema, Pneumomediastinum and Pneumothorax in a Patient with Dermatomyositis

**Published:** 2017-03

**Authors:** Mehdi Bakhshaee, Mohammad Hassan Jokar, Zahra Mirfeizi, Elham Atabati, Somayeh Tarighat

**Affiliations:** 1 *Sinus and Surgical EndoscopicResearch Center, Faculty of Medicine, Mashhad University of Medical Sciences, Mashhad, Iran.*; 2 *Department of Otorhinolaryngology-Head & Neck Surgery, Mashhad University of Medical Sciences, Mashhad, Iran.*

**Keywords:** Dermatomyositis, Pneumomediastinum, Pneumothorax, Polymyositis, Subcutaneous emphysema

## Abstract

**Introduction:**

Spontaneous pneumomediastinum, pneumothorax, and subcutaneous emphysema are rare, but serious complications of inflammatory myopathies and occur more commonly in DM than PM. complications of dermatomyositis (DM) and polymyositis (PM), both of which can be fatal.

**Case Report:**

A 20-year-old woman was admitted with neck pain, dyspnea, cough, and fever. She had been diagnosed with dermatomyositis 21 months prior. A thorax computed tomography (CT) scan revealed ground glass opacities in her lungs, pneumomediastinum, pneumothorax, and subcutaneous emphysema. Despite intensive immunosuppressive therapy, clinical deterioration and radiological progression were observed, ultimately the patient died.

**Conclusion:**

During the care for a patient with dermatomyositis, the otorhinolaryngologist should be cautious of rapidly progressive and fatal neck subcutaneous emphysema. For a patient with dermatomyositis and with normal bronchoscopy and esophagoscopy, the main treatment is control of dermatomyositis with medical therapy. Therefore, a tracheostomy and/or mechanical ventilation may not be necessary.

## Introduction

The prevalence of interstitial lung disease (ILD) varies widely (20-80%) between case series of patients with dermatomyositis (DM) and polymyositis (PM) (1).Occasionally, dermatomyositis or polymyositis-related ILD presents with a rapid onset and progression. Subcutaneous emphysema, pneumothorax, and pneumomediastinum are rare, yet serious, complications of inflammatory myopathies and occur more commonly in DM cases than PM cases (2).

Virtually all patients with DM or PM who develop a pneumomediastinum have interstitial lung disease, and the pneumo- mediastinum is occasionally the clinical presentation of ILD. It is thought that the main cause of pneumomediastinum in patients with DM and PM is architectural distortion of lung tissue due to ILD.Another possible explanation is that vasculopathic lesions within the respiratory tract may result in bronchial or alveolar wall injury and subsequent air leakage (3).

## Case Report

A 20-year-old woman was admitted to our hospital (Imam Reza Hospital, Mashhad, Iran) with neck pain, fever, cough, and dyspnea. She had been diagnosed with dematomyositis 21 months prior. Her symptoms began in March 2014 with polyarthralgia and skin rash. Examination at that time showed presence of heliotrope rash, malar rash, and Gottron’s papules. Muscle forces were intact. Laboratory tests showed a hemoglobin level of 11.9 g/dL (12-16 g/dL), a white blood cell count of 6800/mm^3^ (4000-10000/mm^3^), a platelet count of 261×10^3^/mm^3^ (150-450×10^3^/mm^3^). Blood chemistry showed a glutamate-oxaloacetate transaminase level of 118 IU/L (5-40 IU/L), a glutamate-pyruvate transaminase level of 78 IU/L (5-40 IU/L), a creatine kinase level of 293 IU/L (50-190 IU/L), and an aldolase level of 5.1 IU/ml (＜7.6 IU/ml). 

Urine analysis was normal. Antinuclear antibodies, anti-double stranded DNA, and anti-Jo-1 were all negative. Electromyography was normal. Biopsy of the quadriceps femoris muscle showed no abnormal findings. She was diagnosed with possible DM based on cutaneous manifestations and elevated muscle enzymes. She was treated with prednisolone 50 mg daily and azathioprine 100 mg daily. Two months later, her arthralgia improved, but cutaneous manifestations had not changed. Laboratory tests showed the serum CK level had increased to 727 IU/L. Hydroxychloroquine was added to the previous regime. 

Two months later she was asymptomatic and the serum CK level was normal. Thus, prednisolone was tapered. She felt well until July 2015 when she complained of dyspnea. During chest examination, bilateral fine crackles were heard. Chest x-ray showed bilateral opacities and computed tomography (CT) scan showed bilateral ground glass opacities. Prednisolone dose was increased to 50 mg/day and a cyclophosphamide regimen of 1000 mg per month was initiated. After 2 months, she did not feel better and chest x-ray showed progression of fibrosis. An intravenous immunoglobulin (IVIG) regimen of 400 mg/kg/day for 5 days was prescribed. One month later, she was admitted for neck pain and increased dyspnea. She reported chills and fever for 10 days. Upon physical examination, she was febrile (T=38.2 °C) and her blood pressure was 120/90mmHg. A subcutaneous crepitus around her neck was detected. Muscle power was normal in all four limbs. 

**Fig1:**
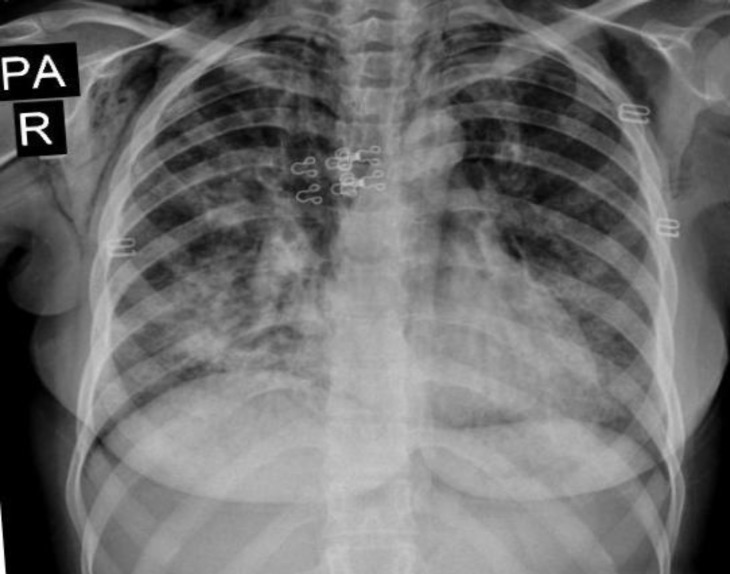
Chest radiograph showing bilateral interstitial and reticulonular changes

**Fig 2:**
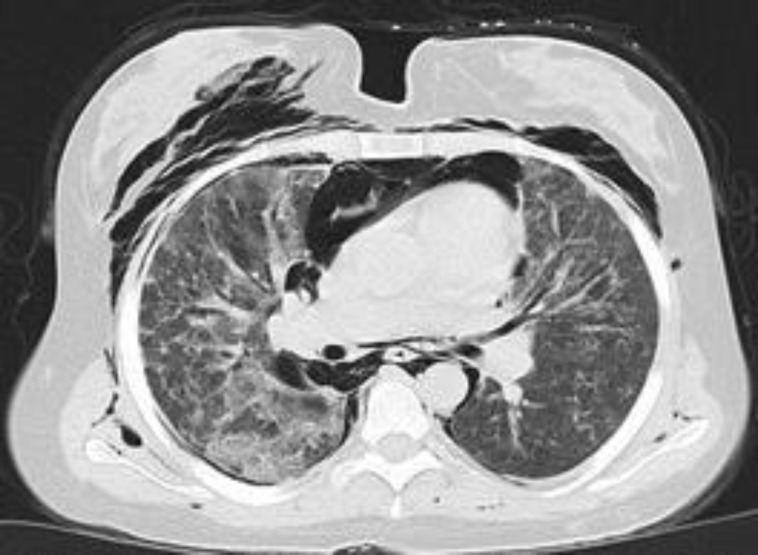
Chest computed tomography scans showing subcutaneous emphysema, pneumomediastinum, pneumothorax, thickening of interlobular septa, and a reticulonodular pattern

Laboratory examination only revealed leukocytosis (23000, PMN 85%) and other laboratory tests (including CK and aldolase) were normal. Chest radiograph and CT scan of the thorax showed a progression lung involvement with subcutaneous emphysema, pneumomediastinum, thickening of interlobular septa, and a reticulonodular pattern (Figs 1,2).

**Fig 3:**
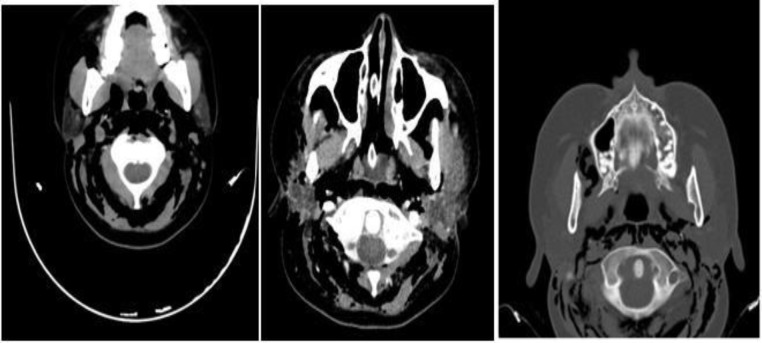
Computed tomography scan of paranasal sinuses showing emphysema in parietal, temporal, and occipital soft tissue and retropharyngeal space

CT scan of paranasal sinuses also showed emphysema in parietal, temporal and occipital soft tissue. Furthermore, emphysema was seen in retropharyngeal space (Fig.3).

The smear and culture of sputum were negative for infectious agents. Flexible bronchoscopy showed severe erythema in bronchial tree, but there was no apparent fistula or rupture. Bronchoalveolar lavage fluid was negative for infectious agents. Echocardiography and esophagoscopy were normal. Intravenous cefteriaxon (1g twice daily) and oral aziothromycin were initiated. On the third day of admission, her shortness of breath worsened. The blood O_2_ saturation was 82%. High-dose steroid pulse therapy (1g IV methylprednisolone for 3 days) and rituximab 500 mg per week for 4 weeks were initiated. Oxygen therapy and complete bed rest were prescribed.

On the 5^th^ day of admission, she transferred to the intensive care unit (ICU) due to severe hypoxemia. She developed bilateral severe pneumothrox. A chest tube was inserted into both pleural spaces. The patient was intubated andmechanical ventilation was initiated. After one week, percutaneous tracheostomy was done.

Ix weeks after immunosuppressive therapy, emphysema and pneumomediastinum were restricted and the chest tube was disconnected (first right, then left). However, the patient died because of severe hypoxemia despite intensive immunosuppressive therapy and mechanical ventilation. 

## Discussion

Dermatomyositis and polymyositis are systemic inﬂammatory diseases affecting skeletal muscles and other organs, including the respiratory system. Lower respiratory tract involvement is still a common cause of morbidity and mortality in DM/PM patients. The most common radiographic finding is a diffuse reticulonodular pattern with patchy ground glass appearance. ILD may cause life-threatening complications (4).

Pneumomediastinum occurs spontaneously or secondary to chest trauma or perforation of trachea, bronchus, or esophagus. Spontaneous pneumomediastinum is usually caused by the rupturing of alveoli due to high intraalveolar pressure. In severe cases of ILD, paracardiac or subpleural blebs can be formed due to the destruction of lung tissue. Rapture of these blebs can lead to leakage of air into the mediastinum, but the precise mechanism is still unknown. Several reports have described mortality rates of 37.5%-52.5% (4-6).

Spontaneouspneumomediastinum, pneumo- thorax, and subcutaneous emphysema are very rare in patients with DM/PM, and there are only several case reports in the medical literature of this occurring (2). It is associated with a poor prognosis. Dermatomyositis complicated by pneumomediastinum is more frequent in men (1,7).

In one study, pneumomediastinum had a prevalence of 8.3% among patients with DM/PM and ILD (8). In a recent retrospective cohort study of 70 DM/PM patients with ILD, pneumothorax or pneumomediastinum was reported in 8.6% of patients, and the 6 patients with pneumothorax or pneumomediastinum died (9).Absence of CK elevation in dermatomyositis appears to be a poor prognosis factor. In a study of 20 cases with slight elevation of CK and without myositis, it was found that the prognoses of these patients were quite poor, and 10 of the patients died due to respiratory failure. But the relationship between DM/PM without serum CK level is still unknown (10,11).

There is no standard treatment for PM/DM lung complications. Cozzani et al. reviewed 55 amyopathic dermatomyositis (ADM) cases with lung involvement and reported that a high dose of systemic glucocorticoids was the first line of therapy in all patients. Immunosuppressive or immunomodulant agents, such as methotrexate, azathioprine, cyclosporine A, cyclophos- phamide, IVIG, mycophenolate mofetil or tacrolimus can be useful (12).

## Conclusion

A rapidly progressive and fatal ILD with subcutaneous emphysema, pneumomediastinum, and pneumothrox is likely. The main treatment is the control of dermatomyositis with medical therapy, however diagnostic procedures including bronchoscopy and esophagoscopy are essential. Tracheostomy and mechanical ventilation may not be necessary because of the severity of subcutaneous emphysema, unless respiratory distress or severe hypoxemia occurs. 
